# Spatial clustering of food insecurity and its association with depression: a geospatial analysis of nationally representative South African data, 2008–2015

**DOI:** 10.1038/s41598-020-70647-1

**Published:** 2020-08-13

**Authors:** Andrew Tomita, Diego F. Cuadros, Tafadzwanashe Mabhaudhi, Benn Sartorius, Busisiwe P. Ncama, Alan D. Dangour, Frank Tanser, Albert T. Modi, Rob Slotow, Jonathan K. Burns

**Affiliations:** 1 Centre for Rural Health, School of Nursing and Public Health, College of Health Sciences, University of KwaZulu-Natal, Private Bag X7, Durban, South Africa; 2 KwaZulu-Natal Research Innovation and Sequencing Platform, Nelson R Mandela School of Medicine, College of Health Sciences, University of KwaZulu-Natal, Durban, South Africa; 3 Department of Geography and Geographic Information Science, University of Cincinnati, Cincinnati, USA; 4 Health Geography and Disease Modeling Laboratory, University of Cincinnati, Cincinnati, USA; 5 Centre for Transformative Agricultural and Food Systems, School of Agricultural, Earth and Environmental Science, University of KwaZulu-Natal, Pietermaritzburg, South Africa; 6 London School of Hygiene and Tropical Medicine, London, UK; 7 School of Nursing and Public Health, College of Health Sciences, University of KwaZulu-Natal, Durban, South Africa; 8 The Lincoln International Institute for Rural Health, University of Lincoln, Lincoln, UK; 9 Africa Health Research Institute, University of KwaZulu-Natal, Durban, South Africa; 10 School of Life Sciences, University of KwaZulu-Natal, Durban, South Africa; 11 Department of Genetics, Evolution and Environment, University College, London, UK; 12 Department of Psychiatry, University of KwaZulu-Natal, Durban, South Africa; 13 Institute of Health Research, University of Exeter, Exeter, UK

**Keywords:** Depression, Epidemiology

## Abstract

While food insecurity is a persistent public health challenge, its long-term association with depression at a national level is unknown. We investigated the spatial heterogeneity of food insecurity and its association with depression in South Africa (SA), using nationally-representative panel data from the South African National Income Dynamics Study (years 2008–2015). Geographical clusters (“hotpots”) of food insecurity were identified using Kulldorff spatial scan statistic in SaTScan. Regression models were fitted to assess association between residing in food insecure hotspot communities and depression. Surprisingly, we found food insecurity hotspots (p < 0.001) in high-suitability agricultural crop and livestock production areas with reliable rainfall and fertile soils. At baseline (N = 15,630), we found greater likelihood of depression in individuals residing in food insecure hotspot communities [adjusted relative risk (aRR) = 1.13, 95% CI:1.01–1.27] using a generalized linear regression model. When the panel analysis was limited to 8,801 participants who were depression free at baseline, residing in a food insecure hotspot community was significantly associated with higher subsequent incidence of depression (aRR = 1.11, 95% CI:1.01–1.22) using a generalized estimating equation regression model. The association persisted even after controlling for multiple socioeconomic factors and household food insecurity. We identified spatial heterogeneity of food insecurity at a national scale in SA, with a demonstrated greater risk of incident depression in hotspots. More importantly, our finding points to the “Food Security Paradox”, food insecurity in areas with high food-producing potential. There is a need for place-based policy interventions that target communities vulnerable to food insecurity, to reduce the burden of depression.

## Introduction

Despite two decades of political advancement since liberation from the apartheid regime and the advent of democracy in 1994, South Africa continues to face intractable poverty^1^, income inequality^2^, and unemployment^3^, that results in many citizens struggling to meet their basic household needs. Consistent with recent global trends^4^, there has also been a disconcerting decline in South African food security, a concept defined internationally as having enough food at all times for an active, healthy life^5^. Since 2011, there has been a growing number of individuals living below the food poverty line (25.2% in 2015 versus 21.4% in 2011^1^), where approximately a quarter of households are exposed to food insecurity in South Africa (which ranged from 21.5% to 23.9% between the years 2010–2015^6^).

Depression, our study outcome of this current investigation, is also a serious health condition that affects 9.8% of South Africans in their lifetime^7^. As a leading cause of disability globally^8^, depression is understood to have the highest negative impact on productivity among all medical conditions^9,10^. Evidence based on financial modelling strongly underpins the assertion that untreated depression can be considered both a health and development challenge for many low- and middle-income countries^11^. In South Africa, workplace-related economic loss from depression is estimated to be 4.9% of gross domestic product (GDP)^12^, a major impediment to economic development for a developing nation, which is further compounded by an additional GDP loss of 5% due to high rates of malnutrition^13^.

Food insecurity and depression are dual public health challenges that are often closely linked. Several systematic reviews confirm a link between food insecurity and mental health^14,15^, with likely mechanisms including both biological processes related to nutritional deficiencies and the psychological stress of having insufficient food^16–18^. Consumption of several nutrients, including vitamin B12 and antioxidants, appear important to prevent depression^19,20^. As a self-reported construct, food insecurity at household- or individual-level also encapsulates an important psychological aspect (i.e. anxiety about restricted food supplies) that is central to the experience of living with food insecurity^21^.

Achieving food security, as part of the United Nation’s Sustainable Development Goal (SDG) 2.1^22^, and in terms of Section 27(1)(b) of the South African constitution^23^, as well as preventing depression onset (SDG 3.4^24^), remain monumental challenges in the light of budgetary limitations to address multiple development priorities. This dilemma warrants targeted strategies to first identify food-vulnerable geographical locations, so as to inform prioritized interventions for the most affected areas (which may have mental health implications, yet to be examined). With some exceptions from Nigeria^25^ and Ethiopia^26^, there are few recent national-level studies from sub-Saharan African (SSA) countries^27^ on the spatial variability of household food insecurity. In this current study, we will use of novel spatial statistical methods to first identify geographical clusters (“hotpots”) of food insecurity, and assess its association with depression, based on panel data from the South African National Income Dynamics Study (SA-NIDS), a unique nationally-representative sample of South Africans, with geographical coordinates for each household. As alluded to previously, the association between food insecurity and depression is well-established at the individual-level^28–30^. Second, we aimed to identify risk of depression onset between individuals living in/outside food insecure hotspot communities over and above the individual effect of household-level food insecurity.

## Methods

Data from the South African National Income Dynamics Study (SA-NIDS) wave 1 (year 2008), wave 2 (2010), wave 3 (2012), and wave 4 (2015) were utilized. As the first panel survey of a nationally representative sample of households in South Africa, the SA-NIDS provides unique insights into population trends in living conditions and well-being that is rarely observed in SSA. The SA-NIDS employs a stratified, two-stage cluster sample design to attain a nationally representative sample of households. In the first stage, 400 of the 3,000 Primary Sampling Units (PSUs) from Statistics South Africa’s Master Sample were selected for inclusion and proportionally allocated, based on the 53 district councils in South Africa. In the second stage, clusters of dwelling units were systematically drawn within each PSU, with two clusters of 12 dwelling units being selected from each. All consenting adult resident household members (≥ age 15) at the selected dwelling units were administered the SA-NIDS questionnaire, its details being described in a published methodological report^31^. For study participants between the ages of 15 and 17, the SA-NIDS obtained written informed consent from a parent and or legal guardian. In minor cases where there were no parents or legal guardians, written informed consent was obtained from caregivers, consistent with South African National Department of Health Guidelines for ethics in health research^32^.

Our study constructed an *incident cohort* based on similar methods utilized in previous studies^33,34^, to better isolate the effect of exposure to food insecurity (i.e. residing in food insecurity hotspot communities) at baseline on subsequent depression onset; thus, reducing the likelihood of reverse causation. The purpose of constructing the incident cohort was to ensure that the observed study participants were free of depression initially; and then to track the risk of depression onset over time between individuals exposed and unexposed to food insecurity. Household food insecurity information was only available for wave 1 (baseline) in the SA-NIDS. Therefore, the incident cohort of our current study consisted of wave 1 participants who screened negative for depression in wave 1 (baseline) and for whom there was a further depression rating score available in any of waves 2–4 (thus participants who screened positive for depression in wave 1 were excluded). We right censored the data, either at the earliest observation at which a participant subsequently screened positive, or at the last observation if the participant did not subsequently screen positive for depression. The SA-NIDS study, approved by the Ethics Committee of the University of Cape Town, obtained written informed consent for all study participants. Our use of SA-NIDS data was approved by the University of KwaZulu-Natal Biomedical Research Ethics Committee (BE 111/14).

## Measures

### Depression

Depression was the main study outcome. Information on depression, based on the ten-item abridged version of the Center for Epidemiologic Studies Depression Scale (CES-D), were obtained from the SA-NIDS Adult questionnaire. The CES-D is a commonly-used psychometric valid/reliable instrument^35,36^ that captures self-reported depression-associated symptoms during the past week. Each of the items has four possible responses in a Likert format, ranging from 0 = rarely/none of the time (less than 1 day) to 3 = almost/all of the time (5–7 days). Depression symptomatology is based on a composite score of the 10 items (Cronbach’s α = 0.75), with a total score of ≥ 10 being classified as a cutoff to signify significant depressive symptoms, consistent with a previous study^37^. Importantly, in this study we use this cutoff to define ‘depression’ as a presentation characterized by significant depressive symptomatology, although this cannot be considered equivalent to a clinical diagnosis of major depressive disorder.

### Household food insecurity

The information on household food insecurity was obtained from wave 1 (2008) of the SA-NIDS Household questionnaire and captured the adequacy of household food needs over the last month. The measure was based on a three-point Likert scale on the adequacy of the food needs, ranging from 1 = less than adequate for household’s needs, to 3 = more than adequate. We generated a final household food insecurity measure (where food adequate/more than adequate = 0 and less than adequate = 1) to be used for the regression analysis (and for generating geographical clustering described immediately below).

### Geographical clusters (“hotpots”) of food insecurity

Geographical clusters (“hotpots”) of food insecurity were identified using Kulldorff spatial scan statistic^38^ implemented in SaTScan software^39,40^. The space permutation model was utilized to identify spatial clusters of households with food insecurity, which were unlikely to have arisen by chance, by testing whether they were significantly adjacent in space. The scan statistical analysis imposed a circular window with varying radii continuously for each global positioning system (GPS) coordinate of the SA-NIDs households located throughout South Africa. The analysis identified a number of distinct potential clusters of affected households with the statistical significance of each being tested using a likelihood ratio test. After a food insecurity hotspot was identified (p < 0.05), its strength within compared with outside the hotspot was estimated using relative risk (RR). Individuals were subsequently either classified as exposed to a food insecurity hotspot (i.e. residing in a household located within a hotspot) or unexposed to a food insecurity hotspot (i.e. residing in a household located outside the hotspots). The SA-NIDS household GPS coordinates were accessed (with permission) from the DataFirst’s Secure Data Centre at the University of Cape Town.

### Statistical analysis

First, a descriptive analysis of the sociodemographic details of the incident cohort was conducted. Second, geographical clusters of food insecurity (i.e. hotspots) were identified (based on the method described above) and the socio-demographic correlates of these ‘hotspot populations’ assessed. Socio-demographic disparities between the hotspot exposed and unexposed were tested using the second-order correction method for survey design^41^ and subsequently converted into F statistics. Third, we investigated the baseline association between residing in food insecure hotspot communities and the likelihood of depression by fitting a generalized linear model^42^ (GLM) based on the *prevalence* cohort (labelled Model 1). The *prevalence cohort*, as opposed to *incident cohort*, included all adults (i.e. 15,630 adults) regardless of depression status at baseline. This analysis was conducted to supplement the results from the above-mentioned second analysis, and to quantify the ‘immediate effect’ of residing in food insecurity hotspot on depression at baseline. Lastly, for the primary focus of our study, we investigated the long-term risk of depression onset due to exposure to hotspots over time based on the *incident cohort* who were depression-free at baseline. For this final analysis, given the repeated measurements of the SA-NID data structure, we fitted two generalized estimating equation^43^ (GEE) regression models. The first (labelled Model 2a) is a model based on variables included in Model 1. The second (labelled Model 2b) is our full model with household food insecurity variable nested within Model 2a. The significance of the nested model that added household food insecurity was tested using Wald test. All regression models were adjusted for sociodemographic variables (e.g. gender, race, age, educational attainment, employment status, income, and urban/rural). Given the nature of the complex survey design in the SA-NIDS, all the analyses involving proportion and regressions were adjusted by post-stratification weight to allow our results to better represent the South African population. The construction of post-stratification weight by SA-NIDS is documented in the published report^44^. All methods were performed in accordance with the relevant guidelines and regulations.

## Results

### Sociodemographic characteristics

Our incident cohort consisted of 8,801 participants who were depression-free at baseline (i.e. year 2008), with the socio-demographic characteristics (Table 1) of the incident cohort indicating that 55.3% (n = 5,169) were female, most were Black African (n = 6,771; 78.6%), and were unemployed (n = 5,300; 56.8%). The largest under-35-age group was 15 to 19 (n = 1,886; 19.9%). The prevalence of food insecurity among the incident cohort was 35.1% (n = 3,284). The number of depression cases among the incident cohort (i.e. depression free at baseline) during the subsequent waves 2–4 were 2,008, 1,330 and 659, respectively.

**Table 1:** Baseline sociodemographic characteristics of incident cohort (N = 8,801 not depressed in Wave 1).

	Overall	
	n	%
**Gender**		
Male	3,632	44.66
Female	5,169	55.34
**Race**		
African	6,771	78.55
Coloured‡	1,409	8.51
Asian/Indian	132	2.66
White	489	10.27
**Age category**		
15–19	1,886	19.92
20–24	1,224	14.1
25–29	861	11.04
30–34	757	10.48
35–64	3,348	37.95
65 +	725	6.51
**Education**		
Less than HS	920	6.8
Completed HS	5,718	61.04
Beyond HS	2,163	32.15
**Employment status**		
Not employed	5,300	56.81
Employed	3,432	43.19
**Household income**		
Lowest 20%	1,602	16.08
Low/middle 20%	2,002	18.59
Middle 20%	1,906	18.66
Middle/high 20%	1,955	22.62
Highest 20%	1,336	24.04
**Residence**		
Rural	4,467	37.92
Urban formal	3,834	53.05
Urban informal	500	9.03

% are adjusted based on post-stratification weight to better match population estimates produced by Statistics South Africa.

*HS* high school.

^‡^The “coloured” is term used by Statistics South Africa^62^, a South African race label that includes children/descendants from Black-White, Black-Asian, Black-Colored, and White-Asian unions^63^.

### Geographical clusters of food insecurity community and its association with baseline depression

**Figure 1:**
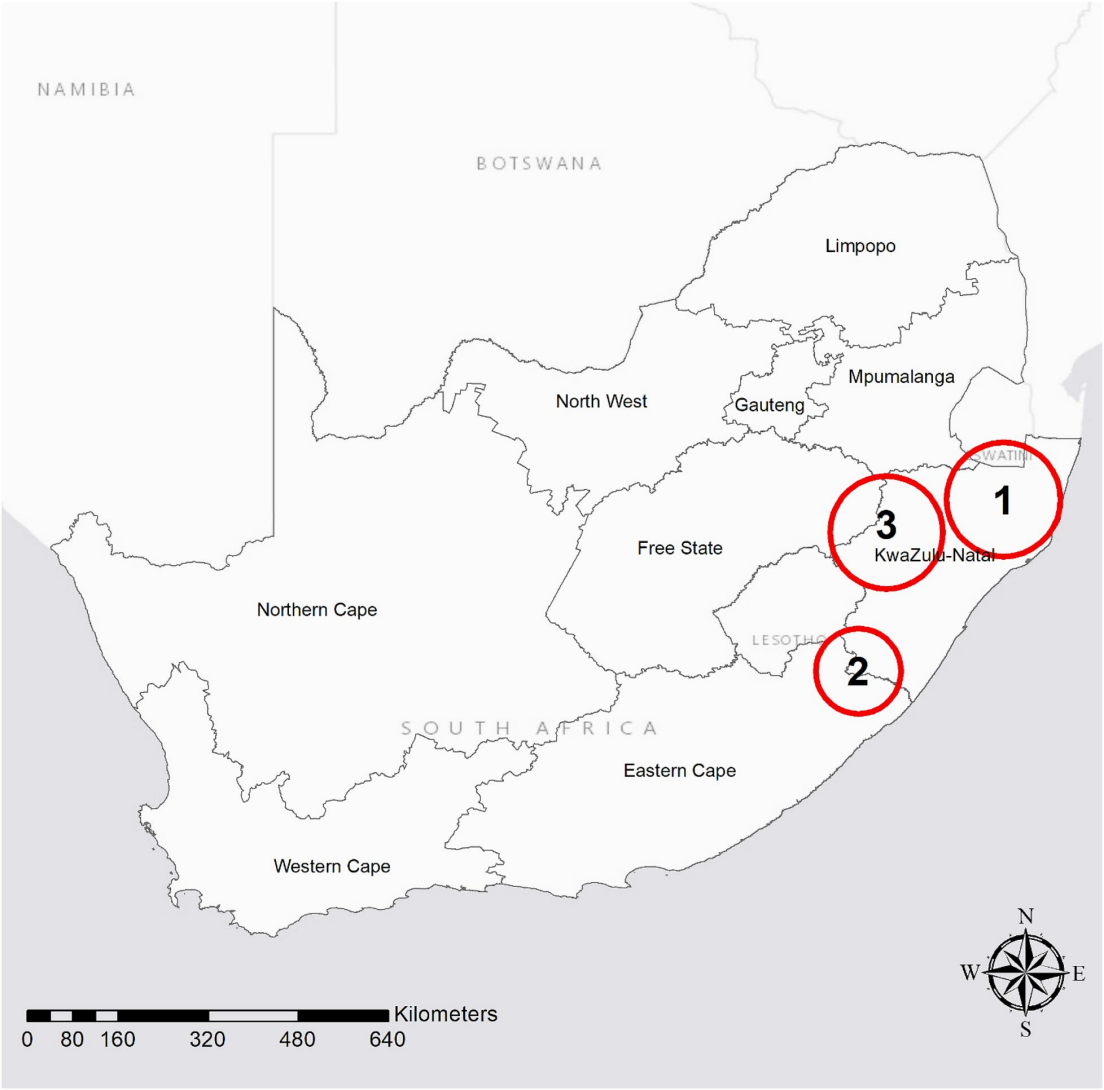
Food insecurity hotspot map of South Africa. Information regarding each cluster number are described in Table 2. Spatial clustering of food insecurity was derived from the SA-NIDS household GPS coordinates accessed (with permission) from the DataFirst’s Secure Data Centre using SaTScan. The map was created using ArcGIS software by Esri version 10.3.

Spatio-temporal scan statistics analysis identified three significant clusters of food insecurity (*p* < 0.05) in South Africa (Fig. 1 and Table 2). There were two clusters in KwaZulu-Natal (KZN) Province and one overlapping both KZN and Eastern Cape. Relative risk of food insecurity within these clusters ranged from 1.34 to 2.21. Among 1,279 individuals residing in food insecure hotspot communities [hereafter refer to as hotspot or hotspot community], 844 reported food insecurity (64.8%). This is in stark contrast to only 2,440 (32.7%) among 7,503 individuals residing outside hotspots reporting food insecurity (*F*(1, 1,061) = 54.86, *p* < 0.01). The significant socio-demographic correlates of residing within a hotspot included: race, being young, having low educational attainment/income, being unemployed, and residing within a rural area (Table 3). We found significantly greater likelihood of depression in individuals residing in hotspot communities [adjusted relative risk (aRR) = 1.13, 95% CI:1.01–1.27] at baseline (Table 4 Model 1) after adjusting for sociodemographic variables, namely gender, race, age, educational attainment, employment status, income, and urban/rural.

**Table 2:** Description of the spatio-clusters of food insecurity in South Africa as depicted in Fig. 1

Cluster	Area (km^2^)	Observed number of cases	Expected number of cases	Strength of the clustering‡	p value
1	25,277	213	101	2.21	< 0.001
2	13,396	103	74	1.41	0.02
3	24,550	140	106	1.34	0.03

^‡^Strength of the clustering estimated as the relative risk of food insecurity within the cluster versus outside the cluster. Areas greater than 10,000 km^2^ are displayed above.

**Table 3:** Baseline sociodemographic characteristics of incident cohort by exposure and non-exposure to food insecurity hotspot community.

				Non-hotspot community					Hotspot community		df_bet_	df_within_	*F*	*p*
				n		%			n	%				
**Gender**														
Male				3,119		92.75			513	7.25	1	1,061.00	0.26	0.61
Female				4,403		92.46			766	7.54				
**Race**														
African				5,503		90.77			1,268	9.23	1.35	1,433.27	13.03	< 0.001
Coloured‡				1,405		99.53			4	0.47				
Asian/Indian				127		96.33			5	3.67				
White				487		99.77			2	0.23				
**Age category**														
15–19				1,547		89.81			339	10.19	4.51	4,789.57	9.20	< 0.001
20–24				1,017		91.64			207	8.36				
25–29				723		91.19			138	8.81				
30–34				671		95.12			86	4.88				
35–64				2,938		93.85			410	6.15				
65 +				626		94.09			99	5.91				
**Education**														
Less than HS				710		86.81			210	13.19	1.8	1912.00	13.09	< 0.001
Completed HS				4,894		92.02			824	7.98				
Beyond HS				1,918		94.88			245	5.12				
**Employment status**														
Not employed				4,409		91.25			891	8.75	1	1,061.00	9.51	< 0.01
Employed				3,045		94.2			387	5.8				
**Household income**														
Lowest 20%				1,199		86.21			403	13.79	3.23	3,429.90	15.40	< 0.001
Low/middle 20%				1,631		88.65			371	11.35				
Middle 20%				1,660		92.17			246	7.83				
Middle/high 20%				1,746		94.8			209	5.2				
Highest 20%				1,286		98.14			50	1.86				
**Residence**														
Rural				3,460		85.88			1,007	14.12	1.89	2009.72	13.24	< 0.001
Urban formal				3,592		96.45			242	3.55				
Urban informal				470		98.05			30	1.95				

% are adjusted based on post-stratification weight to better match population estimates produced by Statistics South Africa.

*HS* high school.

^‡^The “coloured” is term used by Statistics South Africa^62^, a South African race label that includes children/descendants from Black-White, Black-Asian, Black-Colored, and White-Asian unions^63^.

**Table 4:** Regression model assessing the relationship between food insecurity (both hotspot and household) and depression.

	Model 1				Model 2a				Model 2b			
	Prevalence cohort at baseline only				Incident Cohort				Incident Cohort			
	aRR	SE	95% CI		aRR	SE	95% CI		aRR	SE	95% CI	
**Gender**												
[Male]												
Female												
**Race**												
[White]	1.17	0.04	1.10	1.25	1.08	0.05	0.99	1.19	1.09	0.05	0.99	1.19
African	1.99	0.39	1.36	2.92	1.95	0.31	1.42	2.66	1.92	0.31	1.40	2.63
Coloured‡	1.73	0.35	1.16	2.59	1.55	0.26	1.11	2.16	1.55	0.26	1.11	2.16
Asian/Indian	1.62	0.48	0.90	2.91	0.44	0.16	0.22	0.89	0.44	0.16	0.22	0.89
**Age category**												
[15–19]												
20–24	1.42	0.08	1.27	1.59	2.49	0.27	2.01	3.08	2.48	0.27	2.00	3.08
25–29	1.62	0.11	1.41	1.85	2.74	0.30	2.22	3.38	2.74	0.30	2.22	3.38
30–34	1.57	0.12	1.35	1.83	2.80	0.32	2.23	3.51	2.80	0.32	2.23	3.52
35–64	1.84	0.11	1.64	2.08	2.70	0.26	2.24	3.26	2.71	0.26	2.24	3.27
65 +	1.66	0.12	1.44	1.92	3.28	0.38	2.62	4.11	3.29	0.38	2.63	4.13
**Education**												
[Less than HS]												
Completed HS	0.92	0.05	0.83	1.01	0.92	0.06	0.80	1.04	0.92	0.06	0.80	1.04
Beyond HS	0.76	0.05	0.66	0.87	0.70	0.06	0.59	0.83	0.70	0.06	0.59	0.84
**Employment status**												
[Not employed]												
Employed	0.82	0.04	0.75	0.90	0.88	0.05	0.78	0.98	0.88	0.05	0.78	0.98
**Household income**												
[Lowest 20%]												
Low/middle 20%	0.83	0.05	0.74	0.94	0.86	0.06	0.75	0.98	0.86	0.06	0.75	0.99
Middle 20%	0.89	0.05	0.79	0.99	0.76	0.06	0.66	0.88	0.77	0.06	0.66	0.89
Middle/high 20%	0.8	0.06	0.70	0.93	0.73	0.06	0.63	0.84	0.74	0.06	0.64	0.86
Highest 20%	0.65	0.08	0.52	0.82	0.66	0.06	0.55	0.79	0.68	0.06	0.57	0.81
**Residence**												
[Rural]												
Urban formal	1.06	0.08	0.92	1.22	1.29	0.06	1.17	1.41	1.29	0.06	1.17	1.41
Urban informal	1.09	0.11	0.9	1.33	1.31	0.10	1.14	1.51	1.31	0.10	1.13	1.51
**Food insecurity hotspot community**												
[Residing outside]												
Residing inside	1.13	0.07	1.01	1.27	1.15	0.05	1.05	1.26	1.11	0.05	1.01	1.22
**Household food insecurity**												
[Adequate]												
Inadequate									1.13	0.05	1.03	1.23

The “coloured” is term used by Statistics South Africa^62^, a South African race label that includes children/descendants from black–white, black-Asian, black-colored, and white-Asian unions^63^.

*HS* high school, *aRR* adjusted relative risk, *SE* standard error, *CI* confidence interval.

^├^The regression model adjusted based on post-stratification weight (from final observation of the individual panel) to reflect more recent population estimates produced by Statistics South Africa.

### Geographical clusters of food insecurity community and its association on incident depression

The results of the adjusted regression analysis (Table 4 Model 2a) indicated that residing in a hotspot community was significantly associated with higher subsequent incidence of depression (aRR = 1.15, 95% CI: 1.05–1.26). Lastly, the results based on the full model (Table 4 Model 2b) indicated that residing in a hotspot community was significantly associated with higher subsequent incidence of depression (aRR = 1.11, 95% CI: 1.01–1.22). The association persisted even after controlling for multiple socioeconomic factors such as household income (aRR = 0.68, 95% CI: 0.57–0.81) and household food insecurity (aRR = 1.13, 95% CI: 1.03–1.23) under the full model. The addition of household food insecurity to Model 2b was significant using the Wald test (χ = 7.26, df = 1, p < 0.01), suggesting the depression is likely to be caused by household food insecurity and/or residing in a food insecure hotspot community.

## Discussion

Our study investigated the spatial heterogeneity of food insecurity, and its association with incident depression in South Africa, and yielded two significant findings. First, we found a significantly greater incident depression for those residing in a hotspot that had high levels of food insecurity over and above the individual effect of household food insecurity based on incident cohort. This finding points to the likelihood that depression can be associated with either household food insecurity or residing in hotspot communities or both. Second, we found significant differences in the study participant profile, pointing to social vulnerabilities (e.g.[low] household income, employment, and educational attainment) of individuals residing in food insecure hotspot communities. While alleviating depression is often assumed to require psychotherapeutic and pharmacotherapeutic approaches^45^ in low-resource setting, addressing the underlying social causes (e.g. poverty and hunger in the community) may well be called for. Our findings highlight the need to go beyond biomedical approach, and address broader social determinants of depression in many under-resourced communities in South Africa.

To the best of our knowledge, this is the first study in South Africa that has identified the spatial variability of food insecurity at a national scale, with greater risk of incident depression among individuals residing in food insecure hotspot communities. Our findings on the spatial patterns of “hotpots” provide evidence supporting the “Food Security Paradox”; i.e. food insecurity in areas with a high food producing potential^46^. This paradox poses nuanced challenges in devising place-based policy interventions tailored to vulnerable communities. South Africa in general, being regarded as a food secure nation with the means to produce enough staple foods for all individuals^47^, is nonetheless plagued with widespread chronic household food insecurity; this “Food Security Paradox” phenomenon being particularly evident in KwaZulu-Natal Province (KZN) and its border area with the Eastern Cape Province. KZN’s fertile soils are suitable for commercial and small-scale/subsistence farming, being the best-watered province^48^ and home to many large agri-business firms^49^. The province has the country’s highest proportion of agricultural households (23.0%)^50^, with the hotspot communities being located within municipalities with the highest percentage of subsistence-based livelihoods (South African census^51^).

In the 1970s, viewing hunger as a social rather than a technical problem, Moore and colleagues in *Food first: Beyond the myth of scarcity*^52^, documented how the Sahel zone of North Africa exported food to Europe/North America and wealthy African clientele, the amount exceeding that of provided by international food relief in the midst of the famine. The “Food Security Paradox” is also a South African legacy, with apartheid policies replacing adequate and self-sufficient subsistence farming systems with commercial farming, where black South Africans were labor pools, reliant on cash from employment as a basis for attaining food security^47^.

Weaver and Hadley suggest three pathways from food insecurity to mental health problems such as depression namely: nutritional deficiencies with neurobiological consequences; stress generated by “uncertainty in the household ecology”; and perhaps most pertinent to the current South African context, the negative psychological consequences of relative social comparisons of wellbeing within and between communities^14^. Food has significant social functions^53^, and food insecurity may “amplify or magnify relative differences in wellbeing”, being a “particularly honest signal” of relative wealth/income and wellbeing^14^, both between households within and between different communities. There is substantial evidence showing the negative mental health effects of social comparisons in relation to household income inequality^54^. In the context of contemporary South Africa, a country characterised by one of the highest indexes of income inequality globally, households and communities experiencing relative food insecurity (in comparison with their neighbouring households and communities) are likely to experience feelings of inadequacy, shame and ‘social defeat’—all highly correlated with depressive symptoms and disorders in particular^54^.

The question remains regarding what equity policies can address food insecurity that is also closely linked to improved mental health outcomes for socioeconomically vulnerable populations. While market-based policies for improving food security are necessary, there is also a case to be made for well-planned government policies and interventions, particularly when food security is viewed as a public good^55^. Access to food is also a right guaranteed under Section 27(1)(b) of the Constitution of the Republic of South Africa^23^. There is also a case for reducing negative market externality, given that agriculture was the major driver of habitat loss in KZN^56^. Several government options are worth noting^57^, as they have cross-cutting food security and human well-being implications, such as mental health. Acknowledging that it is plausible that government interventions may give rise to unintended consequences that threaten food security, it is also important to explore the provision and restoration of land/property rights of (previously) disenfranchised individuals for subsistence farming. This seems obvious if ending hunger is to be achieved through the production of sufficient and nutritious amounts of food where it is most needed, and not just through social protection mechanisms such as cash transfers/social grants. Currently, the focus of the South African government policy is not on supporting subsistence farmers, but on transitioning them to semi-commercial farming^58^. A land/property rights-based approach to food security may be less dependent on market forces that determine agricultural commodity and individual purchasing power; and may relate more to the restoration of dignity and psychological liberation [given that black South Africans were forcibly removed from their ancestral (fertile) land for whites as part of the Natives Land Act #27 of 1913^59^]. While the discussion of implementation choices in the provision and restoration of land/property rights, with or without compensation, is outside the purview of this current study, it is important to contextualize this research within the socioeconomic and political debate around food, land, and social justice in contemporary South Africa. Finally, we urge prioritized interventions focused on food security and its mental health impacts for individuals residing in hotspot communities in South Africa, and in particular throughout KwaZulu-Natal province and its bordering area with the Eastern Cape. These are the communities that the data suggest are most affected by the “Food Security Paradox” and, therefore, most in need of comprehensive and integrated interventions that deal, not only with food insecurity per se, but also the consequent negative impacts on human wellbeing, as well as the human rights of dignity and equality.

The major limitation of this investigation is the lack of clinical data that would allow for a diagnosis of depressive disorder; and we have therefore utilized a measure of significant depressive symptomatology as a ‘depression’ outcome. Second, food insecurity status was based on self-report, with limited longitudinal measures. Although we assumed the spatial-temporality of food insecure hotspots, we argued, as indicated in the Introduction section, that household food insecurity, although high, remains consistent over time. Notwithstanding these limitations, for the first time, we identified spatial variability of food insecurity at a national scale, with greater risk of incident depression among individuals residing in food insecure hotspot communities, linked directly to a decline in human wellbeing detectable at the population level. The strength of our investigation rests on spatially analyzing GPS data to identify food insecurity patterns, and linking this to the onset of depression at a national-scale; this, to our knowledge, not having been reported previously in studies from SSA. Furthermore, by constructing an incident cohort, we are better able to establish directionality and a causal path from exposure to food insecurity hotspot communities to onset of depression.

Our study highlights cross-cutting SDGs challenges (i.e. 2.1 and 3.4) and comes at a critical juncture when South Africa is grappling with highly contentious land reform issues to address chronic racial inequality^60^ – an issue that is further complicated by climate change^61^. As we acknowledge the important role of food insecurity in mental health outcomes, hunger in the community is also an emotional and ‘distributive justice’ social issue that is linked to the basic human dignity of South Africans previously dispossessed of their land. Addressing symptoms or manifested mental health challenges will require recognition of this deep-rooted (economic) land injustice that undermined human dignity. Our study underscores the need for place-based policy and prioritized interventions that target communities vulnerable to food insecurity to prevent depression and its associated damaging impact on social development in South Africa.
